# A Community-Based Participatory Approach to the Development and Implementation of an HIV Health Behavior Intervention: Lessons Learned in Navigating Research and Practice Systems from Project HAPPY

**DOI:** 10.3390/ijerph17020399

**Published:** 2020-01-08

**Authors:** Rhonda C. Holliday, Romell Phillips, Tabia Henry Akintobi

**Affiliations:** Department of Community Health and Preventive Medicine, Prevention Research Center, Morehouse School of Medicine, 720 Westview Dr SW, Atlanta, GA 30310, USA; rphillips@msm.edu (R.P.); takintobi@msm.edu (T.H.A.)

**Keywords:** HIV prevention, adolescents, community-based participatory research, implementation, complexity

## Abstract

African American young adults continue to be disproportionately affected by HIV/AIDS. The Southern United States has been particularly affected by HIV/AIDS, accounting for 52% of the new HIV diagnoses. Efforts to reduce the burden of HIV among young African Americans are still needed. Project HAPPY (HIV/AIDS Prevention Project for Youth) was developed and implemented using a community-based participatory research (CBPR) model. There were several challenges that arose during implementation of Project HAPPY that included recruitment, partner engagement, and retention. The realities of implementing an HIV prevention project with urban adolescents is discussed in detail and strategies to overcome these challenges, using a CBPR approach are described. The lessons learned from CBPR implementation of Project HAPPY include: (1) Create a feedback loop to receive community input and guidance throughout the life of the project; (2) Periodic community inventory to determine who is providing similar services to avoid saturation; (3) Prepare for Alternative Partner Engagement; (4) Consult (formally and informally) with the Institutional Review Board prior to submitting proposed changes to avoid unnecessary delays in implementation; (5) Select meaningful incentives for your priority population; and (6) Maintain multiple points of contact with community partners to mitigate the effects of staff turnover.

## 1. Introduction

The Centers for Disease Control and Prevention (CDC) reported in 2017 that 40% of high school students in the United States reported having had sex, 30% indicated they had sex during the last three months and almost half did not use a condom [1]. Among those who reported engaging in sex during the last three months, adolescents aged 15 to 19 years have some of the highest reported rates of sexually transmitted infections (STIs) including Chlamydia, Gonorrhea, Syphilis and HIV^1^. Teens and young adults, ages 15–24, also accounted for over 50% of the 20 million new STI cases reported each year [1]. In 2017, Blacks/African Americans accounted for 13% of the US population but accounted for 43% (16,694) of the 38,739 new HIV diagnoses in the United States and dependent areas [1]. In that same year, Black teens and young adults, ages 13–24, represented more than half (52%) of new HIV diagnoses in that age group—the majority of whom were young, gay and bisexual men [1]. Nearly half of young people who are infected with HIV do not know their status [1].

The Southern region of the United States, stretching from as far north as Delaware, southward to Florida, and westward to Texas, is considered the epicenter of HIV/AIDS (as illustrated by CDC). The CDC reported more HIV cases present in the South than any other region of the US. In 2017, the South made up 52% (19,968) of the new HIV diagnoses in the US, which is equivalent to a rate of 16.1 per 100,000 [1]. Southern states accounted for roughly 46% of all people living with HIV in both 2015 and 2016; nearly 47% of all deaths linked to AIDS were in the South [1].

The State of Georgia has not been immune to the increased rates of HIV in the South. In 2015, Georgia nationally ranked sixth for STIs and fifth for HIV [2]. In 2017, the Georgia HIV prevalence rate for those 13 and older was 31.2 per 100,000. Nearly one-quarter (23%) of persons diagnosed with HIV, statewide, were diagnosed with AIDS within 12 months, which is considered a late HIV diagnosis [3]. Moreover, 24% of 9th graders and 36% of 10th graders in Georgia reported that they were sexually active that year [3].

During adolescence, sexual behavioral patterns are developed, and these behaviors can continue into adulthood [4]. It has been suggested that the most appropriate time for HIV/AIDS education is between the ages of 10 and 19, when sexuality and sexual identity are developed. Specifically, prevention education is ideal between the ages of 13 and 16, when rapid hormonal changes take place [4,5]. Adolescent engagement in other risky behaviors, including experimentation with drugs and alcohol, can impair optimal self-efficacy and judgement and lead to the transmission of HIV and other STIs [6,7,8,9].

These statistics highlight that adolescents are not only sexually active but also engage in behaviors that place them at risk for HIV and other STIs. Given the epidemiological evidence of the continued persistence of HIV and other STIs among Black/African American youth in the South, effective interventions for this population are paramount. To address this need, we employed a community-based participatory research (CBPR) strategy to develop and implement an HIV and STI prevention intervention for African American adolescents ages 14–18. The purpose of this paper is to describe the CBPR processes employed and the complex systems navigated in implementing the HIV/AIDS Prevention Project for Youth (Project HAPPY).

### 1.1. Community-Based Participatory Research (CBPR)

Community-based participatory research (CBPR) emphasizes fostering, deployment and sustaining of community–academic partnerships that share leadership in the planning, implementation, evaluation, and dissemination of “innovative, culturally appropriate and evidence-based interventions that enhance translation of research findings for community and policy change” [10,11]. It involves the intentional elevation of community or patient stakeholder groups, among others, capitalizing on their unique strengths and perspectives. Among the advantages of CBPR are neighborhood-campus trust and relationships, improved relevance of research questions, enhanced research recruitment, contextually relevant (thereby, more effective) interventions, increased collaborative research capacities among communities, academic institutions and agencies and changes in the traditionally and historically unequal power dynamics among diverse stakeholder groups [12,13,14,15,16,17]. Outcomes include both statistically significant research results and practically significant responses to both immediate social services needs and the more politically rooted systems that can create health disparities and inequities.

### 1.2. The Morehouse School of Medicine Prevention Research Center Community Coalition Board

The Morehouse School of Medicine Prevention Research Center (Morehouse PRC), funded by the Centers for Disease Control and Prevention (CDC) in 1998, strongly holds to the applied definition of CBPR that is: dynamic; “tailor-made”; focuses on prevention; establishes partnerships between communities and research entities; develops improved interventions that are culturally focused and establishes effective health policies addressing health disparities and inequities. This definition has been applied to a myriad of clinical, social, behavioral, and translational research as well as other collaborative public health training, practice and evaluation initiatives for over 20 years [18,19,20,21,22,23,24,25,26,27,28,29,30,31,32,33,34,35,36,37,38,39]. Corresponding community values research priorities and evaluation criteria were created shortly after the Morehouse PRC was established and have been ratified over time [40,41]. The Community Coalition Board (CCB) has served as the governing body for the Morehouse PRC since 1999. The Board is composed of 23 members representing three-member types: neighborhood residents (16 seats; always in majority number), academic institutions (three seats), and health/social service agencies (four seats). Community representatives hold the preponderance of power, literally putting them at the forefront of all CBPR and related approaches. Board members, including academic, agency, and neighborhood representatives, truly represent the community and its priorities. Academic representatives include the faculty that are frequently engaged in carrying out the research, service or training initiatives affiliated with the Morehouse PRC. Each type of agency was strategically selected due their significance in representing among the social determinants of health including, but not limited to, local health services, education, and affordable housing [42]. The CCB bylaws stipulate that neighborhood representatives will always be in the majority, not academic faculty/staff, on the Board and that the Board Chair, Vice Chair and Secretary will always be neighborhood representatives. Hence, in any vote that pits the neighborhoods against the academics and public health professionals, the neighborhood members would have most votes.

Community representatives of the board are carefully vetted through a systematic process given their priority in research governance. Existing community board members or other respected community/civic leaders are consulted to provide recommendations on those whose reputations and existing leadership may be aligned with the goals and mission of the Center. Interested candidates are asked to complete a profile designed to not only assess their community leadership but also their self-reported perception of how their work aligns with the Center. They are then invited to a meeting with the Center leadership, including the Board Chair, to learn more about the Center, board membership and next steps. Their submitted profile is reviewed and discussed at a subsequent board meeting toward final vote by the entire board membership.

The Center has strategically partnered with the CCB and community leaders to facilitate health research and related interventions informed, in large part, by a community health needs assessment (CHNA) conducted every four years. Each CHNA is conducted to guide development of a community led Morehouse PRC research agenda [17,18]. The Morehouse PRC CHNA process is implemented to (1) collect and analyze qualitative and quantitative data from community stakeholders and secondary data sources to identify the health needs, priorities, and perceptions informing research and intervention implementation; and (2) use recommendations for planning and implementing research projects, disease prevention activities, health promotion outreach, and evaluation initiatives in support of a CBPR agenda.

Between 2012 and 2013, CHNA primary data included both secondary data and surveys developed, pilot-tested and administered by CCB members to 361 community residents. The Morehouse PRC research partner communities (RPCs) are defined by demographic characteristics that have resulted in increased incidence and prevalence of both chronic and infectious disease. These communities are demographically and geographically similar and locally inclusive of five neighborhood planning units represented by 32 census tracts. At the aggregate level, 88% of the RPC residents are young African Americans (median age = 30 years) with low educational attainment (26% of adults have not completed high school) and are ranked among the lowest with respect to a constellation of neighborhood health and quality of life amongst the 25 neighborhoods in the City of Atlanta [39,43]. The top health issues identified through surveys were also among the leading black–white morbidity and mortality disparities and included HIV/AIDS and teen pregnancy. This was the foundation upon which the community determined the health priority to inform an intervention in response. In consultation with the CCB, the researchers and community mutually agreed to address HIV, STIs and teen pregnancy with adolescents who lived in a defined geographical area, leading to the development of HIV/AIDS Prevention Project for Youth (Project HAPPY).

### 1.3. Description of Project HAPPY

Project HAPPY was developed to address behaviors that place adolescents, 14–18, at risk for HIV/AIDS and other STIs, using a CBPR approach. The project was designed to address related gaps in the efficacy, effectiveness and prevention literature among African American youth. The primary aim of the project was to examine the effects of intervention components on condom use, initiation of sexual activity and other risky sexual behaviors.

Project HAPPY consisted of four health education interventions: Becoming A Responsible Teen (BART), an evidence-based behavioral intervention [44], HIV-RAAP, an intervention developed and tested previously by the Morehouse PRC [34], and two new interventions that relied on the use of social media. HIV-RAAP was conceptualized to include a parental arm to increase parent-child communication. The social media interventions consisted of two arms. The first arm (social media A) was an eight-session group level intervention focused on having the participants create messages for social media (specifically Instagram) based on information they learned during the group sessions. The second social media arm (social media B) focused on having teens view and interact with the messages via social media that were created in the first arm (social media A).

## 2. Methods and Materials

Project HAPPY occurred in two phases: formative research and implementation. During the formative research phase, information was gathered to inform intervention creation, adaptation and implementation. The formative research phase included a series of focus groups with both teens and parents, convening of an expert review panel of teens and curriculum adaptations. This phase of the project lasted approximately 12 months. The implementation phase, consisting of four health interventions, was implemented over approximately 36 months.

### 2.1. Participants

Eligibility for the study included being African American, between the ages of 14 and 18 and residing in RPCs served by the Morehouse PRC. Both boys and girls were eligible to participate. Potential participants were informed of the general purpose of the project and screened for eligibility. Once they were deemed eligible, a more in-depth description of the project was presented, and minor assent or participant consent was obtained. The contact information for the parents/guardians was obtained, if they were not present, and parental permission was obtained for those under 18.

### 2.2. Sites

Partnerships were developed with local community-based organizations (CBOs) to conduct recruitment for both the formative and implementation phases and hold educational sessions. Original sites included three Boys and Girls clubs, a YMCA, and a community center located in one of the RPCs. Each site received a stipend for hosting sessions. In addition to the partnerships with CBOs, project staff canvassed the community, visited local shopping centers and attended community events to recruit participants for the implementation phase of the study.

As described earlier, there were four intervention arms. Once participants agreed to participate in the project and assent or consent and parental permission (if needed) were obtained, participants were assigned to one of the intervention arms. Assignment to an intervention arm was based on location. Each arm of the intervention was assigned to a specific RPC. When a participant was recruited, their RPC, based on where they resided, determined which intervention they were assigned to receive.

## 3. Formative Research

### 3.1. Focus Groups

Six focus groups were held with both teens and parents/guardians of teens (five teens and one parent/guardian) to adapt the interventions for the priority population. The focus groups were designed to determine health issues of concern for teens, their HIV and STI prevention knowledge, attitudes regarding HIV and STIs, their HIV and STI prevention sources of information, what they thought teens should learn during the intervention and preferred characteristics related to intervention delivery (messenger, conveying the message, incentives, etc.). The parent/guardian focus group included other more specific questions regarding communication about sexual behaviors and activities with their teens. The focus groups were held in community centers.

### 3.2. Expert Review Panel

Following the focus groups, an expert panel of six teens convened to conduct an in-depth review of each of the proposed curricula. Teens were recruited from local high schools in the recruitment catchment area with the assistance of community members. The teens went through an interview process to assess their level of interest and availability to engage with the project. The expert panel examined curricula language, intervention activities and made recommendations for intervention delivery. They also assisted with the training of community health educators through serving as the test audience and providing feedback on their facilitation skills. The teens’ skill, acumen and enthusiasm resulted in the decision to engage them in the implementation phase. During the implementation phase, their roles as expert panelists transitioned to teen ambassadors, assisting with efforts to provide project information and to recruiting their peers. In preparation for their new role, the teens received a lay training in human research protections using the CIRTification (Community Involvment in Research) program [45,46]. The teens were compensated for their time devoted to project activities

### 3.3. Curriculum Adaptations

Based on the information from the focus groups and the expert review panel, modifications were made to include more interactive activities such as a Jeopardy-like trivia game, making sure the facilitators were closer in age to the teen participants and using Instagram as a delivery modality for the social media intervention. The curriculum adaptations took approximately two months to complete.

Despite careful planning and a CBPR-driven needs assessment and formative research phase, we experienced a series of unanticipated challenges during the implementation of the project that required course corrections and community responsive strategies. These challenges included: (1) recruitment (2) partner engagement and (3) retention. For each challenge, the project staff worked with the CCB and other community stakeholders to develop responsive solutions and implement strategies (see Table 1) as detailed in the sections that follows.

## 4. Implementation Phase: Challenges and Solutions

### 4.1. Partner Engagement

In preparation for the grant submission, the researchers secured letters of support from various community organizations and agencies (N = 5) in the RPCs to assist with recruitment and to serve as intervention sites. Once funding was secured, the investigators visited each partner site to reintroduce the project and solidify the commitment of the site for recruitment and hosting the intervention.

Partner organizational systems change during the approximate 28 months lag between first engagement with the sites to secure their participation and project implementation and required community-engaged sensitivity and response strategies. After completing the formative phase of the research, the investigators found that many of the staff initially engaged at sites had left the organizations. Staff turnover meant that there was often a significant interruption in our recruitment and implementation activities. New staff members were unfamiliar with or unaware of the project and the commitments that were made to host the project for recruiting and implementation.

In addition to staff turnover, some community partners included centers that were independently operated and subsequently taken over by local government. During the preparation of the grant proposal, these community centers had significantly more autonomy over who they chose to partner with for community programming. The local government decided to take a more hands-on approach as we ramped up for the implementation phase of the project. As a result of these changes, we were unable to recruit or hold sessions at two of the community partner sites, despite carefully cultivated relationships during the CBPR planning and formative research phases.

To address these challenges, we employed several strategies. First, we worked to develop new relationships with staff members and reintroduce the project. This often took months to accomplish, given staff members’ schedules and competing priorities. Once relationships were reestablished, the staff member often had to gain approval from higher-level management. Second, our CCB was instrumental in helping us to identify and engage with several potential partner organizations via face-to-face meetings or conference calls. These organizations helped us brainstorm new recruitment strategies and recommended additional community sites to host Project HAPPY. These connections did not always result in recruitment opportunities. For example, we were connected to an organization that would have provided access to several of their community locations. We were able to establish a relationship with a staff member within the organization who was poised to seek approval from higher level management. After approximately two weeks of no communication, we discovered the staff member was no longer with the organization. We subsequently engaged the person who had assumed their duties and had to work to reestablish a relationship and reintroduce the project. In the end, the organization decided not to allow us to use any of their sites for recruitment or hosting sessions. While this connection was not successful, it demonstrates the overall commitment of the CCB to the project.

Another strategy employed was to approach progressive faith-based African American institutions within our priority geographical catchment area. These were churches that were more inclusive of sexual orientations, had youth ministries that discussed sexuality, and participated in events such as World AIDS Day. There were also preexisting connections between Morehouse PRC staff, CCB members and the potential church partners. In our approach, we were careful to explain that our project was designed to provide information for adolescents to make healthy choices and improve their communication skills. Among those who were interested, we were still unable to successfully engage churches due to their prioritization of incorporating bible-based teachings into the curriculum, exclusively promoting abstinence messages, and intention to implement programming that did not focus on sexual behaviors. These modifications would have altered the key components of the intervention, significantly distinguishing it from cohorts implemented at other sites and compromising the intervention fidelity and, thereby, the validity of the research results.

Our final partner engagement challenge was competing with organizations within our larger community who were focused on presenting similar programming. We were especially cognizant and concerned about cross contamination. During implementation, we became aware of five organizations offering programming in our targeted geographical area. This saturation of organizations with similar programming proved to be a challenge for two reasons. First, it limited the number of organizations we could partner with to deliver our intervention. Second, it limited our access to potential eligible participants within the community. One of the first organizations we secured a letter of support from during the development of the grant proposals eventually partnered with the local county health department to deliver content similar to our intervention and for the same target demographic. We also reached out to local medical facilities providing care to teens, but these organizations had their own HIV/STI prevention programming targeting teens.

Despite these challenges, we did experience success with community partners. Our most successful community partners were high schools. Initially, we approached the school system, at the district level, to seek permission to place flyers in the schools advertising our project. After completing the application process, we were ultimately not given permission to advertise the project at neighborhood schools.

At the suggestion of our CCB and high school educators, who expressed a need for the type of education we would be providing, we revised our strategy to seek collaborations with traditional, alternative and charter high schools. First, we met with counselors at each school and provided them with Project HAPPY information. We chose to meet with counselors first because they are student-centered, interacting with individual students on a more personal level. Students often confide in their counselors, providing counselors with information on the needs of the student body. Second, once we had buy-in from counselors, we then met with the administrators at the schools, individually, to seek their support. When we implemented our strategy to recruit and conduct programming in the schools, we found that a local CBO was providing similar programming in several of the schools we contacted, thus we chose to focus on the schools without any programming in our topic area to avoid potential contamination. Ultimately, we were able to partner with three high schools to recruit and hold sessions on their campuses. Each school had nuances with respect time constraints and other contextual parameters. Resultantly we worked with each school to develop a tailored implementation schedule unique to their needs and priorities, without compromising the intervention fidelity. The majority of our participants were from the three schools with which we developed these partnerships. Ultimately, these partnerships addressed our recruitment and retention challenges because students were a captive audience and were more likely to attend all sessions compared to participants, we recruited from community settings.

### 4.2. Recruitment

During recruitment, the investigators discovered that many potential participants did not live in the RPCs but regularly used their resources (schools, community centers, churches, etc.). During the first year of recruitment, project staff interfaced with 140 potential participants who met the age eligibility criteria, but only 22 (15%) lived in the RPC (68% n = 15 enrolled in the study). Once this was discovered, the investigators met with the CCB to develop a strategy in response to this challenge. The local, community-centered and data-based approach of the CCB informed a plan to justify an expanded recruitment strategy. This plan broadened the recruitment criteria to include adolescents who lived, worked or played in the selected geographical area. This plan was presented and approved by the CDC. The assent, consent and parental permission forms were then updated with this language and approved by the IRB (IRB Protocol #670552)

Our teen ambassadors, originally recruited for the expert review panel, also assisted with recruitment. The teen ambassadors informed other teens at their schools about the project and were present as community recruitment events. The teen ambassadors served as liaisons between the research staff and potential participants. They relayed valuable information to potential participants without research staff having to be physically at the school and increased Project HAPPY’s overall presence at the schools. The familiarity of having a peer assisting with recruitment increased the level of comfort of potential participants when communicating about the project. Observations of these interactions indicated that potential participants were more engaged when interacting with project staff, after having interacted with the teen ambassadors. These interactions fostered a level of trust between potential participants and research staff.

### 4.3. Retention

Once we successfully recruited eligible adolescents, completed the enrollment process and administered the baseline survey, they began their matriculation into one of the four intervention arms. We then experienced new challenges, particularly with those we recruited from community sites. Participants usually completed the first two sessions and would not return to complete the remainder of the sessions, negatively affecting our retention efforts. We employed multiple communication strategies (phone calls, emails and text messages) with both adolescents and parents to remind them about upcoming sessions, incentives for both survey completion and session attendance. We found that participants would not respond to any form of communication and stop communicating with project staff. We also had many who would indicate intentions to participate and then not show up. Resultantly, on many occasions, our community health educators would devote time to preparing for sessions only to arrive and find no participants. Through strategic partner engagement and CCB advice, there were three areas that affected our retention and for which we developed responses: incentives and incentive strategy, curriculum length, and transportation. These strategies were developed and employed during the second year of the project implementation and are detailed below.

### 4.4. Incentives and Incentive Strategies

During the formative phase, focus groups and the expert teen panel provided insights into the most appropriate participant incentives and approaches. Project HAPPY provided different incentives to participants for their time. Our original incentives were $15 gift cards for survey completion. During the focus groups and teen panel review, it was suggested that we provide an incentive for session attendance. We proposed holding a raffle for participants for a chance to win a $25 gift card based on the number of sessions attended. We were ultimately unable to implement the raffle due to state laws governing the administration of raffles, which precludes raffles if they are not open to the general public (previously unbeknownst to the researchers and IRB). As the project progressed, we were able to increase the incentives for each survey to a $20 gift card and introduce two additional incentives, $10 gift cards to various stores and eating establishments, for session attendance. Participants could earn a $10 gift card for completing the first four sessions and another $10 gift card for completing the last four sessions. As we continued to experience difficulties with retention, we revisited the incentives that were provided.

One non-fiscal incentive suggestion from high school counselors was to offer community service hours for participation in the project. After consultation with our IRB, we implemented a system to acquire up to 20 community service hours based on session attendance. These hours were documented for each student and provided to their counselors. This proved to be one of the most successful incentive strategies, boosting both recruitment and retention.

### 4.5. Curriculum Length

Three of the four curricula consisted of eight group sessions, each session lasting approximately 90 min. The curricula were designed to be implemented twice per week over a four-week period. During the formative research phase, the investigators listened to the teen expert review panel and made sure activities were interactive, there was plenty of time for discussion and that videos were up to date and used, where appropriate. Despite these efforts, keeping the participants engaged and wanting to return for all eight sessions was challenging. One approach we used to address this challenge was to offer the curriculum over the course of two days—four sessions each day for six hours each day. We provided breakfast, lunch and snacks. This approach was successful because the participants, while committing the same amount of time, did not have to commit over a longer period of time. This decreased the risk of losing participants to competing priorities over a longer period of time. Ultimately, participants only had to commit to two days instead of eight. All of the curriculum content was covered and fidelity to the curriculum was maintained.

### 4.6. Transportation

Based on our original project design with our identified community partners, transportation did not emerge as a barrier to participation. During implementation, transportation challenges did emerge. We experienced parents who were not willing or able to bring their children to all the sessions, despite providing permission for their participation. Additionally, some participants did not have the funds to access the public transportation system. To address this barrier, we offered free passes to use for the local public transit system. We were also cognizant of the importance of hosting sessions at locations accessible by public transportation. Together, the changes in incentives and incentive strategy curriculum length and provision of transportation increased our retention from 24% during the first year of implementation to 65% for the remaining two years of project implementation.

## 5. Discussion

Community engagement in research is not an isolated or siloed exercise but is intrinsically connected to the complexities and concerns of researchers who engage human subjects. The statistical power of a study is enabled, in large part, by the sample size. For high stigma, sensitive health issues like sexual behavior, this is all the more complex. The complexities of such interventions are further amplified when African American youth are the focus, because they represent both higher HIV health disparity burdens and, like other adolescents, are in the midst of establishing identity, negotiation and leadership skills on their journey to adulthood. Inadequate attention to how best to deploy community engaged research and partnership strategies towards successful engagement may result in lower recruitment and retention rates, thereby resulting in a failed study where a researcher cannot statistically demonstrate effects, associations or correlations.

Project HAPPY was not conceived or implemented in a vacuum. It was carefully designed to have continuous community input—from conception through to implementation and dissemination. Despite these efforts, Project HAPPY experienced challenges, specifically related to recruitment, partner engagement and retention that we addressed through CBPR and other contextually responsive approaches. We offer the following lessons and advice to researchers, community partners and practitioners involved in CBPR work with adolescents around sensitive topics.

### 5.1. Create a Feedback Loop

Continuously seek community input and guidance from the priority population and those that have a stake in their well-being. Community input can help researchers address issues critical to the implementation of the project. Communities are dynamic entities and are impacted by changes in policies, particularly marginalized communities. These changes can have a significant impact on project implementation strategies. Seeking community input throughout the implementation of an intervention, from trusted stakeholders, can help a researcher course correct, through responding to new realities in programming, governance and preferred ways to engage and retain interest.

### 5.2. Conduct a Periodic Community Inventory

Regularly take inventory of who is addressing the issue in the community to avoid oversaturation or duplication of intervention approaches. Although the community may decide the health issue, there are inter- and intra-system dynamics that come to bear in the success of approaches developed. The interaction between a CBPR-driven, validated and evidence-based intervention system and the community context system in which it is ultimately delivered can conflict. These create implementation challenges based on parallel, evolving systems that are both attempting to be responsive. A continuous inventory of who is providing similar services to youth and adolescents in the community is critical and necessary, as the health and welfare of youth are frequently positioned as a litmus test for a community’s health. In our case, there was also an emphasis placed on HIV prevention by the local government given the epidemic’s impact in the county. As a result, some of the neighborhoods where our project was located were also targeted/prioritized for enhanced services. This greatly impacted the community partnership strategies initially planned and ultimately revised in response.

### 5.3. Prepare for Alternative Partner Engagement

Explore alternative ways to access potential partners serving your priority population. We initially approached the local school board to gain access to advertise our project and were subsequently denied. Through discussion with our CCB and community stakeholders, we were able to identify contacts within schools who had the autonomy to make decisions about the programming they offered. Counselors, administrators, and parent liaisons proved to be critical allies when attempting to access schools as recruitment and implementation sites. Identifying key stakeholders within a system who have a vested interest in the well-being of the priority population is important. These stakeholders can help to navigate systems, overcoming barriers and identify facilitators to project implementation.

### 5.4. Consult (Formally and Informally) with the Institutional Review Board

Consult with your IRB prior to submitting formal proposed changes once you encounter complex systems requiring implementation adjustment. Consultation can provide feedback on what may or may not be acceptable according to state laws and federal regulations. Additionally, when a new procedure is proposed by the community, prior to submission of changes, consulting with your IRB can provide additional guidance and suggestions for protocol changes. We believe that due to the vulnerable population status of youth/adolescent populations in complex research and practice systems, this strategy is even more important to demonstrate a data-based and CBPR-driven approach to attend to the special needs of this population. This strategy also demonstrates the intent of the research team to hold themselves accountable to the nuances and sensitivities necessary to being responsive to the optimum youth engagement strategies that ensure the least risk and maximum benefits to both they and their families.

### 5.5. Select Meaningful Incentives

Identify incentives that are uniquely suited for adolescents still in high school. The adoption of providing community service hours was well received by the participants and proved to be a better fit than the solely monetary incentive strategy initially planned during the proposal development and formative phase. This was an incentive that we had not previously considered, nor was it something we discovered during our formative phase. It is important to think outside the box and listen to suggestions that may be well suited for your prioritized youth/adolescent audience. Crafting an incentive strategy that is meaningful to the participants is vitality important and may also prove to be cost effective. Community service hours were of no cost to the project but proved to be more valuable to the participants than gift cards. If we had not asked and listened to our stakeholders, we may not have discovered and implemented this strategy that was an important factor in increasing retention.

### 5.6. Maintain Multiple Points of Contact with Partners

Establish relationships with more than one point of contact within a community organization and remain in regular contact. Given the staff turnover that can occur in community organizations, this is particularly important for youth-serving organizations. These organizations are understandably very protective of programming designed to serve adolescents due to their vulnerable and impressionable status. This process can be quite a daunting task and may be filled with frustration and disappointment if the broader, complex system of leaders in research and practice that may serve as barriers or facilitators to project success are not strategically considered, at the onset.

Further, the time lag between research proposal submission and grant award can be several months or longer—during which, time change within the partner organizations are inevitable. Therefore, research success requires attention to community organization systems, with relationships maintained year round. Develop materials that quickly and clearly distill the project and how the organization agreed (or will be potentially engaged) early and in anticipation for change. Further, a pre-developed well-crafted email, one-pager or flyer can go a long way in helping to quickly and concisely explain your youth-focused project and its importance to the community-serving organization.

The processes described in this report are designed to acknowledge the realities of a unique CBPR intervention as well as the complex communities in which it was integrated. It is designed to contribute to practical steps for researchers on the importance of nurturing and understanding the nature of work with community organizations, both large and small. Community connections are not just forged during the grant-writing process, they must be continually developed. The reality of community organization system, that holds an important stake in the success of research initiative system, is that it lives, evolves and responds, year- round, rather than being based on that official “start” and “stop” dates of a research project.

Despite these complex intervention system realities, our ability to pivot and respond to time-critical feedback from the CCB allowed us to reach the youth we sought to engage. We recruited 431 teens and enrolled 246 (57%) into the project. Among them, 178 (72%) completed all sessions.

## 6. Conclusions

Attention to the art and science of community engagement, in general, and for special populations like those of the HAPPY project is a unique complex intervention case study, comprehensively detailed through this report. CBPR often reflects how real-world implementation meets the traditional tenets of rigorous research. Flexibility is paramount when listening to the community involved in the intervention and does not have to comprise intervention fidelity. We intentionally engaged with the community and our stakeholders in ways that were beyond what was written in the grant proposal. Anchored by our CBPR philosophy and process, we were successfully able to develop and implement the intervention, cultivate and maintain new and existing relationships with community stakeholders and partners, and empower the community via capacity building (i.e., training our teen ambassadors and certifying them in human subjects training, providing sexual health education training to teachers and providing participants with community service hours). Without these modifications, our recruitment and retention rates would have failed, likely forfeiting this multi-year investment of time and relationship building. Providing the community with tangible benefits, particularly communities that have historically not experienced benefits with regards to research participation, is vitally important. Researchers should thoughtfully consider and incorporate the voice of the communities throughout the research process, creating wins for the researchers and, more importantly, wins for the community.

## Figures and Tables

**Table 1:** Research/Practice Systems Challenges and Solutions.

Implementation Processes	Research/Practice System Challenge(s)	Solution(s)
Recruitment	1. Participants met the age eligibility criteria, but did not meet the Research Partner Community (RPC) residence criteria	1a. Consulted with the Community Coalition Board (CCB) and the Institutional Review Board (IRB) to expand eligible geographic area based on real-time participant daily/weekly engagement in RPC organizations
		1b. Consulted with the CCB and IRB to include participants who used resources in the PRCs (i.e., schools, community centers, churches)
Partner Engagement	1. Staff turnover at partner sites and changes in partner site management	1a. Developed new relationships with new staff. 1b. CCB helped identify and engage several new potential partner organizations
	2. Duplication of services by other community organizations led to saturation	2. Strategically developed relationships, at the suggestion of the CCB and high school education, with schools’ representative champions to stand-up implement phase
Retention	1. Incentives and incentive strategies	1a. Increased fiscal incentives for each survey from a $10 gift card to a $20 gift card 1b. Added two $10 gift cards for session attendance 1c. Integrated non-fiscal incentives through integration of 20 community service hours per the recommendation of high school counselors
	2. Curriculum length	2. Condensed while commitment from four weeks to two-days, when possible
	3. Transportation	3. Provided passes for use on local public transportation system
